# Barriers to Implementation of Perinatal Death Audit in Maternity and Pediatric Hospitals in Jordan: Cross-Sectional Study

**DOI:** 10.2196/11653

**Published:** 2019-03-06

**Authors:** Yousef Khader, Mohammad Alyahya, Anwar Batieha

**Affiliations:** 1 Department of Public Health and Community Medicine Jordan University of Science and Technology Irbid Jordan; 2 Department of Health Management and Policy Jordan University of Science and Technology Irbid Jordan

**Keywords:** perinatal death, quality of health care, cause of death, Jordan

## Abstract

**Background:**

Perinatal death audit is a feasible and cost-effective quality improvement tool that helps to improve the quality of health care and reduce perinatal deaths. Perinatal death audit is not implemented in almost all hospitals in Jordan.

**Objective:**

This study aimed to assess health professionals’ attitude toward perinatal death auditing and determine the main barriers for effective implementation of perinatal death auditing as perceived by health professionals in Jordanian hospitals.

**Methods:**

A cross-sectional study was conducted among health professionals in 4 hospitals in Jordan. All physicians (pediatricians and obstetricians) and nurses working in these hospitals were invited to participate in the study. The study questionnaire assessed the attitude of health professionals toward perinatal death audit and assessed barriers for implementation of perinatal death audit in their hospitals.

**Results:**

This study included a total of 84 physicians and 218 nurses working in the 4 selected maternity hospitals. Only 35% (29/84) of physicians and 36.2% (79/218) of nurses reported that perinatal death audit would help to improve the quality of prenatal health care services to a great or very great extent. Lack of time was the first-mentioned barrier for implementing perinatal death audit by both physicians (35/84, 42%) and nurses (80/218, 36.7%). Almost the same proportions of health professionals reported inadequate patient information being documented in hospital records as a barrier. Lack of a health information system was the third-mentioned barrier by health professionals. Fear of having conflicts with the family of the dead baby was reported by almost one-third of physicians and nurses. Only 28% (23/83) of physicians and 16.9% (36/213) of nurses reported that they would like to be involved in perinatal death audit in their health facilities.

**Conclusions:**

Health professionals in Jordan had poor attitude toward perinatal death audit. The main barriers for implementing perinatal death audit in Jordanian hospitals were lack of time, inadequate patient information being documented in hospital records, and lack of health information systems.

## Introduction

Perinatal death audit is a feasible and cost-effective quality improvement tool that helps to improve the quality of health care and reduce perinatal deaths [1]. Perinatal death audits are implemented to generate accurate perinatal data, determine medical and nonmedical causes of perinatal deaths, identify appropriate interventions to address these causes, and improve the quality of services. Countries that have implemented perinatal death audit have achieved a significant decrease in perinatal deaths. Studies have shown that perinatal death audit was associated with a 30% reduction in perinatal mortality [2].

Perinatal death audits have been used widely in high-income countries [3,4]. However, they are less frequently used in low- and middle-income countries where 98% of perinatal deaths take place [5]. Few studies have assessed the attitudes of health care providers about perinatal death audits, the challenges facing their implementation, and the barriers for implementing perinatal death audits. One study [6] showed that physicians have positive attitudes toward the death audit and reported that it is a good quality-of-care indicator in the hospital, which are valuable and necessary to improve the quality of health services. However, inadequate patient information in hospital records, lack of time for health care providers, high turnover of health professionals, fear of blame, lack of a national policy for perinatal audit, and lack of training were identified as important barriers for implementing death audit [7-9].

In the late 1990s, the neonatal mortality rate in Jordan fell from 19 to 15 per 1000 live births and remained relatively constant as Jordan transitioned into the new millennium [10]. Jordan is one of the many countries in the world that failed to achieve the Millennium Development Goal 4’s target [11-13]. This is particularly because of the lack of effective planning and monitoring of health services. On the other hand, the United Nations International Children’s Emergency Fund (UNICEF)–funded study “Perinatal and Neonatal (PNN) Mortality study in Jordan” [13] showed that a large proportion (74%) of neonatal deaths were preventable and only 37% of neonatal deaths received optimal health care. The study highlighted the need for strengthening the essential newborn care and improving the quality of maternal and neonatal health care. Other studies in Jordan showed that barriers to access maternal and neonatal care, low social status of women, poverty, and inequality were major determinants of PNN deaths especially in rural and remote areas in Jordan [14,15]. Moreover, the influx of Syrian refugees has a negative impact on economic, social, and health development and has stressed the country’s health system [16].

Previous studies in Jordan strongly recommended establishing and implementing perinatal death audits in hospitals to improve the quality of services and decrease perinatal deaths. In Jordan, most hospitals have perinatal death review committees. However, these committees are not functional, and perinatal death audits have not yet been implemented in health facilities. To build an effective perinatal death audit, it is important to understand health professionals’ attitude toward perinatal death audit and their perception of barriers and challenges for proper perinatal death audit implementation. Therefore, this study aimed to assess health professionals’ attitude toward perinatal death auditing and determine the main barriers for effective implementation of perinatal death auditing as perceived by health professionals in pediatric hospitals in Jordan. Moreover, the study aimed to determine whether health professionals’ attitude and their perception of the main barriers differ according to gender, profession, and years of experience.

## Methods

### Study Design

A cross-sectional study was conducted in 3 public hospitals and 1 teaching hospital, 3 hospitals from the north (Al-Mafraq Pediatrics Hospital, Princess Rahma Hospital, and King Abdullah University Hospital) and 1 from the south of Jordan (Al-Karak Hospital). Princess Rahma Pediatric Hospital (120 beds) and Al-Mafraq Pediatrics Hospital (108 beds) are the only pediatric referral public hospitals in Jordan. Out of the 2 teaching hospitals in Jordan, we selected King Abdullah University Hospital (683 beds), which is affiliated with Jordan University of Science and Technology (JUST) and serves approximately 1 million inhabitants in the north of Jordan. Al-Karak Hospital is the largest and the main hospital that provides pediatric services in the south of Jordan, with 125 beds. All physicians (pediatricians and obstetricians) and nurses working in these hospitals were invited to participate in the study, and those who agreed were interviewed using face-to-face structured interview by trained nurses. The study was approved by the institutional review board at JUST.

### Study Questionnaire

The first part of the study questionnaire collected information about health professionals’ age, gender, and years of experience. The second part of the questionnaire included 2 main questions to assess the perception of health professionals about perinatal death review: “To what extent a perinatal death review committee would help to improve the quality of prenatal healthcare services?” and “To what extent a perinatal death review committee would help to reduce perinatal deaths?.” The possible responses for each question were “not at all,” “to a small extent,” “to some extent,” “to a moderate extent,” “to a great extent,” and “to a very great extent.” For the purpose of analysis, the responses “to a great extent” and “to a very great extent” were pooled together in 1 category to indicate great extent.

The third part of the questionnaire assessed the barriers for implementation of perinatal death audit in their hospitals. Health professionals were presented with a list of potential barriers that were identified from the relevant literature [17-29]. Moreover, they were asked to report any barrier that is not mentioned in the list.

### Statistical Analysis

Data were analyzed using IBM SPSS version 20. Data were presented using percentages for categorical variables and means and SDs for continuous variables. The differences between proportions were tested using chi-square test. A *P* value of less than .05 was considered statistically significant.

## Results

### Participants’ Characteristics

This study included a total of 84 physicians and 218 nurses working in the 4 selected maternity hospitals. More than half (44/84, 53%) of physicians were females, and almost all nurses were females. Their age ranged from 17 to 62 years, with a mean (SD) of 31.1 (6.5) years. Their years of experience ranged from 1 to 35 years, with a mean (SD) of 7.6 (6.5) years and median of 5.0 years. All selected hospitals had a nonfunctional perinatal deaths review committee.

### Attitude Toward Perinatal Death Audit

Only 35% (29/84) of physicians and 36.2% (79/218) of nurses reported that perinatal death audit would help to improve the quality of prenatal health care services to a great or very great extent (Table 1). Similarly, 32% (27/84) of physicians and 38.5% (84/218) of nurses stated that perinatal death audit would help to reduce perinatal deaths (Table 1). However, 12% (10/84) of physicians and 21.6% (47/218) of nurses reported that perinatal death audit would not help to improve the quality of prenatal health care services, and an almost similar proportion reported that the perinatal death audit would not help to reduce perinatal deaths.

Table 2 shows the participants’ responses on whether perinatal death audit would help to improve the quality of prenatal health care services and reduce perinatal deaths to a great or very great extent according to gender, profession, and years of experience. The attitude of health professionals toward perinatal death auditing did not differ significantly according to gender, profession, and years of experience.

**Table 1:** Health professionals’ attitude toward perinatal death audits in maternity and pediatric hospitals in Jordan.

Attitude toward perinatal death audit		Health professionals		Total (N=302), n (%)
		Physicians (n=84), n (%)	Nurses (n=218), n (%)	
**The extent to which a perinatal death audit would improve the quality of prenatal health care services**				
	Not at all	10 (12)	47 (21.6)	57 (18.9)
	To a small extent	7 (8)	13 (6.0)	20 (6.6)
	To some extent	12 (14)	29 (13.3)	41 (13.6)
	To a moderate extent	26 (31)	50 (22.9)	76 (25.2)
	To a great extent	20 (24)	64 (29.4)	84 (27.8)
	To a very great extent	9 (11)	15 (6.9)	24 (7.9)
**The extent to which a perinatal death audit would help to reduce perinatal deaths**				
	Not at all	10 (12)	40 (18.3)	50 (16.6)
	To a small extent	16 (19)	13 (6.0)	29 (9.6)
	To some extent	7 (8)	25 (11.5)	32 (10.6)
	To a moderate extent	24 (29)	56 (25.7)	80 (26.5)
	To a great extent	22 (26)	69 (31.7)	91 (30.1)
	To a very great extent	5 (6)	15 (6.9)	20 (6.6)

**Table 2:** Participants’ responses on whether perinatal death audit would help to improve the quality of prenatal health care services and reduce perinatal deaths to a great or very great extent according to gender, profession, and years of experience.

Variable		Perinatal death audit would help to improve the quality of prenatal health care services			Perinatal death audit would help to reduce perinatal deaths			
		Total	n (%)	*P* value		Total	n (%)	*P* value
**Gender**				.30				.38
	Male	42	12 (29)			42	13 (31)	
	Female	258	95 (36.8)			258	98 (38.0)	
**Profession**				.78				.30
	Physician	84	29 (35)			84	27 (32)	
	Nurse	218	79 (36.2)			218	84 (38.5)	
**Years of experience**				.72				.62
	≤5	158	58 (36.7)			158	56 (35.4)	
	>5	144	50 (34.7)			144	55 (38.2)	

### The Main Barriers for Implementation of Perinatal Death Audit

The main barriers for implementing perinatal death audit in the hospitals were lack of time, inadequate patient information being documented in hospital records, lack of health information system, and fear of having problems with the family of the dead baby (Table 3). Lack of time was the first-mentioned barrier by both physicians (35/84, 42%) and nurses (80/218, 36.7%) for implementing perinatal death audit. Almost the same proportions of health professionals reported inadequate patient information being documented in hospital records as a barrier. Lack of a health information system was the third-mentioned barrier by health professionals. Fear of having conflicts with the family of the dead baby was reported by almost one-third of physicians and nurses. Fear of legal problems and the sensitivity between the concerned physicians and nurses were reported as barriers for effective implementation of perinatal death audit by 25.8% (78/302) and 23.2% (70/302) of health professionals, respectively. Having difficulties in ensuring confidentiality and not trained to conduct perinatal death were reported by almost one-tenth of physicians and nurses. Health professionals’ frequent turnover was the least-mentioned barrier. Physicians were significantly more likely to report “not trained to conduct perinatal death review” as a barrier compared with nurses (17/84, 20% vs 17/218, 7.8%; *P* value=.004). The participants’ responses in regard to the perceived barriers for effective implementation of perinatal death audits in hospitals in Jordan did not differ significantly according to gender and years of experience.

### Intention to Be Involved in Perinatal Death Audit

Only 28% (23/83) of physicians and 16.9% (36/213) of nurses reported that they would like to be involved in perinatal death audit in their health facilities. More than half of the physicians (48/83, 58%) and 63.8% (136/213) of nurses stated that they would probably like to be involved in perinatal death audit if it is implemented in their health facilities. The intention of health professionals to be involved in perinatal death audit did not differ significantly according to gender, profession, and years of experience (Table 4).

**Table 3:** The main barriers for effective implementation of perinatal death audits in hospitals in Jordan as perceived by health professionals.

Main barriers for effective implementation of perinatal death audits	Health professionals		Total (N=302), n (%)	*P* value
	Physicians (n=84), n (%)	Nurses (n=218), n (%)		
Lack of time	35 (42)	80 (36.7)	115 (38.1)	.48
Inadequate patient information being documented in hospital records	34 (41)	77 (35.3)	111 (36.8)	.45
Lack of health information system	33 (40)	75 (34.4)	108 (35.8)	.48
Fear of having problems with the family of the dead baby	29 (35)	74 (33.9)	103 (34.1)	.97
Fear of medico-legal problems	25 (30)	53 (24.3)	78 (25.8)	.41
Sensitivity between the concerned physicians and nurses	18 (21)	52 (23.9)	70 (23.2)	.76
Difficulty in ensuring confidentiality	16 (19)	24 (11.0)	40 (13.2)	.10
No need for the death review	7 (8)	30 (13.8)	37 (12.3)	.27
Not trained to conduct perinatal death review	17 (20)	17 (7.8)	34 (11.3)	.004
Health professionals’ frequent turnover	5 (6)	8 (3.7)	13 (4.3)	.58

**Table 4:** The participants’ responses to whether they would you like to be involved in perinatal death audit if it is implemented in their health facilities.

Variable		Would you like to be involved in perinatal death audit in your health facility?				*P* value
		Definitely yes, n (%)	Probably yes, n (%)	No, n (%)		
**Gender**						.07
	Male	14 (33)	22 (52)	6 (14)		
	Female	45 (17.9)	160 (63.5)	47 (18.7)		
**Profession**						.10
	Physician	23 (28)	48 (58)	12 (14)		
	Nurse	36 (16.9)	136 (63.8)	41 (19.2)		
**Years of experience**						.35
	≤5	28 (17.9)	103 (66.0)	25 (16.0)		
	>5	31 (22.1)	81 (57.9)	28 (20.0)		

## Discussion

This study showed that only one-third of health professionals had reported that perinatal death audit would help to improve the quality of prenatal health care services to a great or very great extent. As perceived by health professionals, lack of time, inadequate patient information being documented in hospital records, and lack of a health information system were the first 3 mentioned barriers for implementing perinatal death audit. The attitude of health professionals toward perinatal death auditing and their intention to be involved in perinatal death audit did not differ significantly according to gender, profession, and years of experience. The perceived barriers for effective implementation of perinatal death audits in hospitals in Jordan did not differ significantly according to gender and years of experience.

The causes of perinatal deaths in Jordan need to be quickly addressed if the Sustainable Development Goals target is to be met. To increase the survival of babies, it is essential to identify the causes of perinatal deaths and their contributing factors and improve the quality of services. This can be achieved by effective implementation of perinatal death audit in Jordan hospitals. The audit process offers a chance to learn from critical situations in the management of maternity and neonatal care. Health care providers are urged to modify their care to better practice once there is detection about the poor practices that lead to these problems [17]. The capability to respond efficiently to recommendations acknowledged through audits is crucial to reducing deaths.

Over the past few decades, Jordan has made substantial progress in improving maternal, neonatal, and infant health. However, there are still challenges to achieving the third Sustainable Developmental Goal. Existing references indicate that the majority of perinatal deaths are preventable. Jordan is now ready for the next step toward eliminating preventable perinatal deaths. A vital component of any elimination strategy is a continuous surveillance system that not only tracks the number of deaths but also provides information about the underlying contributing factors and how they should be addressed. Stillbirths and neonatal deaths surveillance “J-SANDS” and auditing system is a model of such a system.

Although literature supports the fact that perinatal death audit strongly contributes to the avoidance of perinatal deaths, a relatively small proportion of health professionals (27/84, 32% of physicians and 84/218, 38.5% of nurses) stated that perinatal review audit would help to reduce perinatal deaths. This finding reflects the poor awareness of the value of perinatal death audit among health professionals. The main barriers to perinatal death audit implementation in our study included lack of time for health care providers, inadequate patient information in hospital records, lack of health information system, and fear of having problems with the family of the dead baby. Lack of time by health professionals was the first-mentioned barrier in our study. This barrier has been reported in other studies [18-20]. In a study conducted in Uganda, the majority of respondents reported that the main challenge to conduct death review was heavy workload with fewer staff [21]. To overcome this barrier, perinatal death audit should be included in job descriptions of health professionals [19]. The management should also support perinatal death review as one of the health professionals’ duties and as a part of their daily work.

Inadequate patient information in hospital records was the second-mentioned barrier. This barrier was also reported as a barrier to completing audit successfully in many studies in Malawi, Tanzania, and Uganda [18,22-25]. Inadequate information hinders the ability of health professionals from assessing the causes of deaths.

Lack of an electronic health information system was the third-mentioned barrier. Many hospitals in Jordan do not have the capacity to process the limited available data to capture deaths, assign causes of deaths, and identify the avoidable factors. One study in Jordan showed that only 14% of neonatal deaths are registered and reported to the Department of Civil Registration because Jordan relies on paper-based systems to register and report births and deaths. None of the hospitals in Jordan report stillbirths. Lack of an electronic health information system and lack of a centralized database for compiling audit results makes data interpretation and identification of avoidable factors difficult to create actionable recommendations. Electronic health information system and centralized database for compiling audit, registering births and deaths, and assigning causes of deaths should be developed and implemented. Electronic platforms may pose an initial additional financial burden, although they may save time and money in the long term [26].

Fear of blame including loss of face among peers and potential legal ramifications have been shown to be important deterrents to conducting perinatal death audit in other studies [27]. To ensure successful implementation, having participants agree to a code of conduct for review meetings, establishing a no-blame environment, and ensuring confidentiality insofar as it is possible contribute to an environment where audit is more likely to be successful [27].

Health professionals’ frequent turnover was the least-mentioned barrier in our study. However, this was shown as an important barrier in other studies [28]. Consistent with other studies, not being trained on perinatal death audit was one of the mentioned barriers [29]. Unlike our studies, previous studies reported other barriers such as the lack of a national policy, strategy, and guidelines for perinatal audit [7]. However, the availability of a policy alone does not guarantee the success of the implementation.

One of the main limitations of this study is that that the findings cannot be generalized to all hospitals of Jordan because 2 of the selected hospitals were pediatric hospitals, 1 was a teaching hospital, and the fourth hospital was a public hospital in the south of Jordan. Moreover, our findings are limited only to public and teaching hospitals as we did not include private hospitals. Another limitation is that the sample of health professionals is small to conduct subgroup analysis. In conclusion, health professionals in Jordan had a poor attitude toward perinatal death audit. The main barriers for implementing perinatal death audit in Jordanian hospitals were lack of time, inadequate patient information being documented in hospital records, lack of a health information system, and fear of having problems with the family of the dead baby. Training activities are needed to increase the awareness of health professionals about the value of perinatal death in improving the quality of services and perinatal deaths. An electronic health information system and centralized database for compiling audit, registering births and deaths, and assigning causes of deaths should be developed and implemented.

## Abbreviations

JUST: Jordan University of Science and Technology
PNN: perinatal and neonatal
UNICEF: United Nations International Children’s Emergency Fund
